# Relevance of Anthropometric Measurements in a Multiethnic Obesity Cohort: Observational Study

**DOI:** 10.2196/27784

**Published:** 2021-05-13

**Authors:** Rahila Bhatti, Usama Warshow, Mona Joumaa, Mariam ElSaban, Faisal A Nawaz, Amar Hassan Khamis

**Affiliations:** 1 Mediclinic Parkview Hospital Dubai United Arab Emirates; 2 Mohammad Bin Rashid University of Medicine and Health Sciences Dubai United Arab Emirates

**Keywords:** anthropometrics, body mass index, cardiovascular health, comorbidities, liver disease, obesity, overweight, type 2 diabetes mellitus, visceral fat, waist circumference, weight loss, weight management

## Abstract

**Background:**

The prevalence of obesity is increasing worldwide, and the Middle East is not an exception to this increasing trend. Obesity increases the risk of multiple metabolic complications, such as diabetes mellitus. Measurement of obesity has primarily relied on the BMI to identify risk; however, both bedside and office-based anthropometric measures of obesity can provide more detailed information on risk.

**Objective:**

This study aimed to investigate the prevalence of obesity-related diseases in a multidisciplinary weight management population and to determine its relationship with obesity anthropometric indices.

**Methods:**

This cross-sectional study was conducted at Mediclinic Parkview Hospital (Dubai, the United Arab Emirates). In total, 308 patients have been evaluated from January to September 2019 as part of a multidisciplinary weight management program. Key demographics, anthropometrics, and clinical data were analyzed using SPSS (version 25, SPSS Inc).

**Results:**

Our cohort of 308 patients included 103 (33%) males and 205 (67%) females of 38 nationalities. The mean age of the cohort was 41 (SD 9.6) years, with a median BMI of 34.5 (IQR 6.7) and 33.7 (IQR 7.8) for males and females, respectively. The mean waist circumference (WC) was 113.4 (SD 23.3) cm and 103.5 (SD 16.2) cm, fat percentage was 33.7% (SD 11.6%) and 45% (SD 6.8%), fat mass was 41 (SD 15.2) kg and 41.1 (SD 14.1) kg, and visceral fat mass was 6.5 (SD 3.2) kg and 3.1 (SD 1.8) kg for males and females, respectively. There was a strong correlation between BMI and WC (*r*=0.65 and *r*=0.69 in males and females, respectively; *P*=.01) and visceral fat (*r*=0.78 and *r*=0.90 in males and females, respectively). Furthermore, visceral fat was significantly associated with WC in both sexes (*r*=0.73 and *r*=0.68 in females and males respectively; *P*=.01). Furthermore, WC was significantly associated with a risk of diabetes, hypertension, and nonalcoholic fatty liver disease.

**Conclusions:**

BMI and WC are the most representative measures of obesity in our population and correlate with abdominal adiposity– and obesity-related diseases. Further studies are required to assess the benefits of these measures during weight reduction interventions.

## Introduction

The prevalence of overweight and obesity has increased worldwide, as defined by the BMI. In the United Arab Emirates, the obesity prevalence rate reported in 2016 was 29.9% [1,2]. There is a paralleled increase in the incidence of metabolic-related conditions, particularly type 2 diabetes, metabolic syndrome, and nonalcoholic fatty liver disease (NAFLD) [2]. Although BMI is the most used measure to classify at-risk individuals and to assign treatments, it does not reflect abdominal obesity, which is a surrogate marker for visceral adiposity. Visceral adiposity is a strong predictor of cardiovascular [3] and metabolic risk [3].

Waist circumference (WC) is a simple anthropometric parameter to assess abdominal adiposity in clinical practice. WC is strongly associated with cardiovascular mortality [3,4]. Therefore, it has been recommended to determine the WC in conjunction with BMI to assess the metabolic risk in accordance with a 2008 expert consultation report of the World Health Organization [5].

The aim of our study was to validate the utility of anthropometric measures other than BMI in the assessment of obesity and their relationship with metabolic conditions including diabetes, hypertension, and NAFLD in a cross-sectional cohort of individuals with overweight and obesity.

## Methods

### Patients and Study Design

This cross-sectional observational study was conducted at Mediclinic Parkview Hospital (Dubai, the United Arab Emirates) between January and September 2019. A total of 308 patients enrolled in the hospital’s multidisciplinary weight management program were included in this study. Patients in the program included those who were referred from outpatient Mediclinic cluster clinics, outpatient and inpatient consultations at Mediclinic Parkview Hospital, or those who self-referred to the program. Patients were then directed by the bariatric coordinator of the program to the dietician or physician as required. After initial assessments, the patients were provided individualized weight management plans including a dietetic plan, exercise, behavioral therapy, medication, or bariatric surgery as indicated. The weight management team included a dietician, an endocrinologist, a gastroenterologist, a bariatric surgeon, and a psychologist. Patients requiring bariatric surgery were discussed in multidisciplinary team meetings before surgery with close postsurgical follow-up.

Data were collected from electronic medical records of Bayanaty (InterSystems IRIS). The data were collected in four categories: demographic data, anthropometric measures, laboratory measurements, and clinical disease and risk factor status.

Demographic data included age, gender, and nationality. Anthropometric measures included height, weight, BMI, fat mass, body fat percentage, visceral fat mass, WC, hip circumference, and waist-hip ratio (WHR). Laboratory measurements included glycated hemoglobin (HbA_1c_), renal function including creatinine and estimated glomerular filtration rate, liver function tests including aspartate transaminase and alanine transaminase activity, lipid profile including cholesterol, triglyceride, and low- and high-density lipoprotein (HDL) cholesterol. Clinical variables included the presence of diabetes, hypertension, polycystic ovarian syndrome, dyslipidemia, and NAFLD. 

### Obesity-Related Metabolic Risk Factors

Four metabolic syndrome components were included in the analysis: hypertension (systolic/diastolic blood pressure of ≥130/85 mmHg or taking drug treatment for hypertension), hyperglycemia (HbA_1c_=6.5% or taking diabetes treatment), hypertriglyceridemia (≥150 mg/dL or 1.7 mmol/L or taking drug treatment for elevated triglycerides), and low HDL cholesterol (<40 mg/dL or 1.0 mmol/L in men and 50 mg/dL or <1.3 mmol/L in women or taking drug treatment).

### Obesity Parameters and Anthropometric Variables

BMI was defined as the body weight divided by the square of the height in meters (kg/m^2^). World Health Organization recommendations were used to categorize individuals by weight as follows: healthy weight (BMI=20.0-25.0), overweight (BMI=25.0-29.9), and obese (BMI≥30.0) [6].

WC was defined as the measurement midway between the lowest rib and the iliac crest by using a flexible tape measure. Hip circumference was measured at the level of the greater trochanters to the nearest millimeter by using a flexible tape measure. WHRs were obtained by dividing the WC by the hip circumference. Although many have recommended different ethnicity-based WC cut-offs, there is insufficient evidence to recommend different cut-offs for individuals of European rather than those of Middle Eastern or African ethnicities [7]. Therefore, for these ethnicities, “the cut-off WHRs were 94.0-101.9 cm and 80.0-87.9 cm for men and women with overweight and >102 cm and >88 cm for men and women with obesity, respectively. Although there is evidence that WC cut-offs for obesity in Asian populations vary from those of Europeans [8], this population constituted a small proportion of Asians; hence, separate cut-offs were not assigned. Men with a WHR of <0.90, 0.90-0.99, and ≥1.0 were classified as having a normal weight, overweight, or obesity, respectively, and women were classified in the same categories on the basis of a WHR of <0.80, 0.80-0.84, and ≥0.85, respectively.

Anthropometric data were collected using a body composition analyzer (Seca GmbH), which uses bioelectric impedance analysis to determine the body fat mass, body fat percentage (% fat and % fat mass), and the visceral fat ratio. The normal fat percentage for women is 21%-35% and that of men is 8%-24%. A normal visceral fat ratio is >1.2 and >2.1 for women and men, respectively [9].

### Statistical Analysis

Data were entered in a computer, using SPSS for Windows (version 25.0, SPSS Inc). Frequency tables, the measure of percentage, and the measures of tendency and dispersion were analyzed as descriptive data. Categorical variables were cross-tabulated to examine the independency between variables; for such variables, the chi-square test or Fisher-exact test was used as appropriate. The Kolmogorov–Smirnov test was used to test the normality of continuous variables. The Mann–Whitney *U* test was used to compare the means between 2 groups if the normality was not confirmed, while the 2-tailed *t* test was used for normal data per groups. A *P* value less than .05 was considered significant.

### Ethics Statement

Ethics approvals were obtained from the local Mediclinic Institutional Research Board and the Dubai Scientific Research Ethics Committee, Dubai Health Authority.

## Results

A total of 308 patients who had participated in the weight management program were included in the study, and all had either overweight or obesity. A larger proportion of females participated in the weight management program (n=205, 67%) with a mean age of 41 years. The enrolled patients represented 38 different nationalities. The majority were of Middle Eastern and North-East African ethnicity (n=166, 54%), and the remaining were of European and Asian ethnicity (n=80, 26% and n=58, 19%, respectively).

Table 1 shows the prevalence of obesity-related metabolic conditions (Table 1). Diabetes was prevalent among almost half of the male patients with obesity (n=28, 49%) compared to only 8% among patients with overweight. Among females, dyslipidemia, NAFLD, and hypertension were significantly more prevalent among patients with obesity than among those with overweight.

**Table 1:** Comparison of complications between patients with overweight and those with obesity by gender (N=308).

Complications		Male patients (n=103)					Female patients (n=205)			
		Overweight, n (%)	Obesity, n (%)	*P* value		Overweight, n (%)		Obesity, n (%)	*P* value	
**Diabetes**					.008					.28
	No	11 (92)	29 (50)			37 (79)		63 (72)		
	Yes	1 (8)	28 (49)			10 (21)		24 (28)		
**Hypertension**					.36					.006
	No	7 (58)	28 (48)			40 (91)		60 (71)		
	Yes	5 (42)	31 (53)			4 (9)		25 (29)		
**Polycystic ovarian syndrome**					N/A^a^					.24
	No	11 (100)	42 (100)			34 (79)		62 (74)		
	Yes	0	0			8 (19)		22 (26)		
**Dyslipidemia**					.11					.02
	No	5 (42)	10 (18)			22 (60)		39 (53)		
	Yes	7 (58)	47 (82)			15 (41)		35 (47)		
**Nonalcoholic fatty liver disease**					.25					.045
	No	3 (60)	19 (46)			20 (95)		39 (72)		
	Yes	2 (40)	23 (55)			1 (5)		15 (28)		

^a^N/A: not applicable.

Laboratory parameters that are used to define diabetes, NAFLD, dyslipidemia, and chronic kidney disease were compared between male and female patients with overweight and those with obesity (Table 2). HbA_1c_ values were not significantly different between the 2 groups (*P*=.30 for men and *P*=.20 for women). Mean alanine transaminase levels, a marker of NAFLD, were higher in patients with obesity than in those with overweight, but this difference was only significant in female patients (21.9 U/L vs 34.3 U/L, respectively, *P=*.05). In terms of lipid parameters, triglyceride levels were found to be significantly higher in patients with obesity than in those with overweight among men (2.4 mmol/L vs 1.2 mmol/L, respectively; *P=*.01). In women, HDL cholesterol levels were lower in patients with overweight than in those with obesity (1.5 mmol/L vs 1.2 mmol/L, respectively; *P=*.004).

**Table 2:** Laboratory parameters compared between male and female patients with overweight and those with obesity (N=308).

Parameter		Male patients (n=103)					Female patients (n=205)			
		Overweight, mean (SD)	Obesity, mean (SD)	*P* value		Overweight, mean (SD)		Obesity, mean (SD)	*P* value	
**HbA_1c_ levels**					.30					.20
	HbA_1c_, %	5.6 (0.6)	6.2 (2.0)	N/A^a^		5.8 (0.7)		5.5 (1.0)	N/A	
	HbA_1c_, mmol/mol	38 (0.6)	45 (2.0)	N/A		39 (0.7)		37 (1.0)	N/A	
Creatinine, µmol/L		94.8 (21.0)	82.2 (20.0)	.20		62.3 (8.0)		65.1 (8.0)	.20	
Estimated glomerular filtration rate, mL/min/1.73 m^2^		86.3 (20.0)	93.7 (21.0)	.40		105.1 (12.0)		98.8 (18.0)	.10	
Aspartate transaminase, U/L		35.8 (23.0)	31.7 (23.0)	.70		21.8 (7.0)		30.7 (29.0)	.70	
Alanine transaminase, U/L		31.2 (11.0)	47 (39.0)	.40		21.9 (13.0)		34.3 (30.0)	.05	
Cholesterol, mmol/L		5.9 (0.3)	9.4 (29.0)	.70		5.2 (2.0)		10.4 (33.0)	.50	
Triglycerides, mmol/L		1.2 (0.3)	2.4 (1.0)	.01		1.1 (0.5)		1.6 (0.8)	.07	
Low-density lipoprotein cholesterol, mmol/L		3.4 (2.0)	6.4 (21.0)	.70		3.6 (1.0)		3.6 (1.0)	.90	
High-density lipoprotein cholesterol, mmol/L		2.3 (2.0)	1.9 (6.0)	.70		1.5 (0.4)		1.2 (0.3)	.004	

^a^N/A: not applicable.

Table 3 shows the anthropometric measurements of all the patients. Males had significantly higher weight, height, WC, BMI, and visceral fat mass (6.5 kg vs 3.1 kg in males and females, respectively; *P*<.001), while females had a higher fat percentage than males, but this difference was not significant (45% vs 37%, respectively; *P*=.40).

**Table 3:** Gender-specific anthropometric characteristics of patients (N=308).

Characteristics	Males (n=103), mean (SD)	Females (n=205), mean (SD)	*P* value
Weight, kg	110.2 (24.0)	89.0 (20.0)	<.001
Height, cm	171.0 (36.0)	155.4 (34.0)	<.001
BMI, kg/m^2^	34.5 (7.0)	33.7 (8.0)	.006
Waist circumference, cm	113.4 (23.0)	103.5 (16.0)	<.001
Hip circumference, cm	127.0 (12.0)	124.2 (15.0)	.39
Waist-hip ratio	0.9 (0.2)	0.8 (0.1)	.78
Fat mass, %	41 (15)	41 (14)	.86
% Fat	37.0 (6.6)	45.0 (6.8)	.43
Visceral fat, kg	6.5 (3.2)	3.1 (1.8)	<.001

In order to determine the relationship among anthropometric measures, multiple correlation analysis was conducted with the matrix of correlation shown in Table 4. This revealed a strong correlation between BMI and WC (females: *r*=0.65, males: *r*= 0.69; *P*<.001) and visceral fat (females: *r*=0.78, males: *r*=0.90). Furthermore, visceral fat mass was significantly associated with WC in both genders (females: *r*= 0.73, males: *r*=0.68; *P*<.001). Unsurprisingly BMI, weight, and height were strongly correlated with one another because they were interdependent variables.

**Table 4:** Matrix of correlation of the measurements of the anthropometric indicators^a^ of obesity.

Indicators	BMI	Waist circumference	Visceral fat mass	% Fat	Hip circumference	Waist-hip ratio
BMI	—^b^	0.69^c^	0.90^c^	0.34^c^	0.89^c^	0.06
Waist circumference	*0.65* ^c^	—	0.68^c^	0.43^c^	0.85^c^	0.42
Visceral fat mass	*0.78* ^c^	*0.73* ^c^	—	0.63^c^	0.75^c^	0.52^d^
% Fat	*0.18* ^c^	*0.30* ^c^	*0.48* ^c^	—	0.48^d^	0.58^d^
Hip circumference	*0.53* ^c^	*0.65* ^c^	*0.72* ^c^	*0.52* ^c^	—	0.14
Waist-hip ratio	*0.38* ^c^	*0.54* ^c^	–*0.13*	*0.40* ^c^	–*0.23*^d^	—

^a^Values of *r* for females are shown in italics.

^b^—: not applicable.

^c^*P<*.001.

^d^*P*<.05.

## Discussion

### Principal Findings

This observational study shows that anthropometric measurements in combination with BMI, particularly WC, can provide more detailed information on metabolic risk. Our findings have confirmed higher rates of obesity-related factors in individuals with obesity, which is consistent with all existing data [10]. Further, the study has clearly shown a strong relationship between waist circumference and estimated visceral fat mass through body composition analysis, thus emphasizing the routine inclusion of these measures in the assessment of such patients. Our results are similar to those of several other studies showing a strong association between metabolically active visceral fat and cardiovascular risk factors [4,11].

The prevalence of obesity in this study was much higher than that estimated nationwide in the United Arab Emirates (70% vs 34%, respectively) [12] as the weight management program would have enrolled more individuals with obesity. In unselected populations in the United Arab Emirates, the estimated prevalence of obesity was reported to be 34% in 2016 [13]. The multinational, multiethnic composition of the study population renders our findings more generalizable to other populations. Our study participants were of 38 different nationalities, as Dubai is a multicultural city with individuals of >200 nationalities [14].

The BMI definition of obesity does not account for different phenotypes of obesity, in terms of fat distributions and the difference between subcutaneous and visceral adiposity. Particularly, central visceral adiposity is more strongly predictive of metabolic risk factors. Accordingly, our data show that WC and visceral fat mass are significantly correlated with each other. Therefore, this simple and inexpensive measurement should supplement BMI in defining obesity and metabolic risk with potential implications on treatment allocations [15]. Kamadjeu et al [16] reported similar results in a cohort from Cameroon with respect to the burden of diabetes baseline data. It is notable that an increase in abdominal visceral adiposity is reflected by WC and is related to an increased cardio-metabolic risk [3]^.^ WHR had a weak correlation with other anthropometric measures in our cohort.

Furthermore, there are gender differences in the rates of metabolic-related disorders. Our data show higher rates of NAFLD, dyslipidemia, and diabetes in females with obesity but could not be linked to anthropometric variables, particularly WC and the visceral fat ratio. This is likely owing to the small numbers of individuals in some of the groups.

The findings of our study have important implications in the assessment of obesity in clinical practice, as they reinforce the use of anthropometrics as indicators of obesity. The International Atherosclerosis Society and International Chair on Cardiometabolic Risk working group have also published a consensus statement on visceral obesity in March 2020. It is recommended to use the WC value as a critical target for reducing adverse health risks for both men and women [17]. Recent Canadian guidelines for obesity in adults also recommend the measurement of WC in addition to BMI to identify individuals with increased visceral adiposity and adiposity-related health risks [18].

### Strengths and Limitations

The limitations of our study are its sample size and the cross-sectional assessment of anthropometric parameters. More studies are needed to analyze the implications of longitudinal anthropometrics on the occurrence of metabolic-related conditions and the effect of different weight management interventions in modifying this risk.

In summary, this study demonstrates a strong correlation between conventional obesity measures and anthropometric measures, particularly the WC. It highlights the importance of using anthropometrics such as WC as a measure of obesity, especially as it is an easy-to-use and inexpensive clinical tool.

### Conclusions

In conclusion, our study shows that BMI and WC are the most representative measures of obesity in our population and correlate with visceral adiposity and obesity-related diseases. This study highlights the importance of incorporating anthropometrics in the clinical assessment of patients with obesity to further determine their metabolic risk. Further studies are required to assess the benefits of these measures during weight reduction interventions.

## Abbreviations

HbA_1c_: glycated hemoglobin
HC: hip circumference
HDL: high-density lipoprotein
NAFLD: nonalcoholic fatty liver disease
WC: waist circumference
WHR: waist-hip ratio
